# Supplementary material for: Deciphering AKI in Burn Patients: Correlations between Clinical Clusters and Biomarkers
Source: Int J Mol Sci. 2024 Jun 20;25(12):6769. doi: 10.3390/ijms25126769 (PMC11204051; doi:10.3390/ijms25126769)
Supplement: Supplementary file 1 [file ijms-25-06769-s001.zip › ijms-3035364-supplementary.pdf]

Supplementary material

Deciphering AKI in Burn Patients: Correlations between Clinical Clusters and Biomarkers

Shin Ae Lee1, Dohern Kym,1,2*, Jaechul Yoon1,2, Yong Suk Cho1,2, Jun Hur1,2, Dogeon Yoon2

1 Department of Surgery and Critical Care, Burn Center, Hangang Sacred Heart Hospital, Hallym University Medical Center, 12, Beodeunaru-ro 7-gil, Youngdeungpo-gu, Seoul, Korea, 07247

2 Burn Institutes, Hangang Sacred Heart Hospital, Hallym University Medical Center, 12, Beodeunaru-ro 7-gil, Youngdeungpo-gu, Seoul, Korea, 07247

*Corresponding author

Department of Surgery and Critical Care, Burn Center, Hangang Sacred Heart Hospital, College of Medicine, Hallym University 12, Beodeunaru-ro 7-gil, Youngdeungpo-gu, Seoul, Korea, 07247

Tel. 82-2-2639-5446, Fax. 82-2-2678-4386, E-mail: dohern@hallym.or.kr

Contents

STROBE Statement—Checklist of items that should be included in reports of cohort studies 3

Table S1. Creatinine Level Change by Hospital Days ... 6

Figure S1. Trajectory of Creatinine for all patients by Hospital days ... 6

Table S2. Adjusted Odds Ratios for AKI Based on Various Predictors Controlling for Patient and Disease Factors ... 7

Table S3. NRI IDI for Predictors of AKI by Cluster ... 9

Table S4. IDI for Predictors of AKI by Cluster ... 11

Table S5. Net Benefit Comparison for AKI Prediction by Decision Curve Analysis ... 13

STROBE Statement—Checklist of items that should be included in reports of cohort studies

	Item No	Recommendation	Checked
**Title and abstract**	1	(*a*) Indicate the study's design with a commonly used term in the title or the abstract	Yes
		(*b*) Provide in the abstract an informative and balanced summary of what was done and what was found	Yes
**Introduction**			
Background/rationale	2	Explain the scientific background and rationale for the investigation being reported	Yes
Objectives	3	State specific objectives, including any prespecified hypotheses	Yes
**Methods**			
Study design	4	Present key elements of study design early in the paper	Yes
Setting	5	Describe the setting, locations, and relevant dates, including periods of recruitment, exposure, follow-up, and data collection	Yes
Participants	6	(*a*) Give the eligibility criteria, and the sources and methods of selection of participants. Describe methods of follow-up	Yes
		(*b*) For matched studies, give matching criteria and number of exposed and unexposed	Yes
Variables	7	Clearly define all outcomes, exposures, predictors, potential confounders, and effect modifiers. Give diagnostic criteria, if applicable	Yes
Data sources/ measurement	8*	For each variable of interest, give sources of data and details of methods of assessment (measurement). Describe comparability of assessment methods if there is more than one group	Yes
Bias	9	Describe any efforts to address potential sources of bias	No
Study size	10	Explain how the study size was arrived at	Yes
Quantitative variables	11	Explain how quantitative variables were handled in the analyses. If applicable, describe which groupings were chosen and why	Yes
Statistical methods	12	(*a*) Describe all statistical methods, including those used to control for confounding	Yes
		(*b*) Describe any methods used to examine subgroups and interactions	Yes
		(*c*) Explain how missing data were addressed	Yes

		(*d*) If applicable, explain how loss to follow-up was addressed	NA
		(*e*) Describe any sensitivity analyses	Yes
**Results**			
Participants	13*	(a) Report numbers of individuals at each stage of study—eg numbers potentially eligible, examined for eligibility, confirmed eligible, included in the study, completing follow-up, and analysed	Yes
		(b) Give reasons for non-participation at each stage	Yes
		(c) Consider use of a flow diagram	Yes
Descriptive data	14*	(a) Give characteristics of study participants (eg demographic, clinical, social) and information on exposures and potential confounders	Yes
		(b) Indicate number of participants with missing data for each variable of interest	Yes
		(c) Summarise follow-up time (eg, average and total amount)	NA
Outcome data	15*	Report numbers of outcome events or summary measures over time	Yes
Main results	16	(*a*) Give unadjusted estimates and, if applicable, confounder-adjusted estimates and their precision (eg, 95% confidence interval). Make clear which confounders were adjusted for and why they were included	Yes
		(*b*) Report category boundaries when continuous variables were categorized	Yes
		(*c*) If relevant, consider translating estimates of relative risk into absolute risk for a meaningful time period	Yes
Other analyses	17	Report other analyses done—eg analyses of subgroups and interactions, and sensitivity analyses	Yes
**Discussion**			
Key results	18	Summarise key results with reference to study objectives	Yes
Limitations	19	Discuss limitations of the study, taking into account sources of potential bias or imprecision. Discuss both direction and magnitude of any potential bias	Yes
Interpretation	20	Give a cautious overall interpretation of results considering objectives, limitations, multiplicity of analyses, results from similar studies, and other relevant evidence	Yes
Generalisability	21	Discuss the generalisability (external validity) of the study results	Yes
**Other information**			
Funding	22	Give the source of funding and the role of the funders for the present study and, if applicable, for the original study on which the present	Yes

article is based	

*Give information separately for exposed and unexposed groups.

Note: An Explanation and Elaboration article discusses each checklist item and gives methodological background and published examples of transparent reporting. The STROBE checklist is best used in conjunction with this article (freely available on the Web sites of PLoS Medicine at http://www.plosmedicine.org/, Annals of Internal Medicine at http://www.annals.org/, and Epidemiology at http://www.epidem.com/). Information on the STROBE Initiative is available at http://www.strobe-statement.org.

Table S1. Creatinine Level Change by Hospital Days

Group	Characteristic	**HD 1**, N = 463 (28.0%)	**HD 8**, N = 452 (27.3%)	**HD 15**, N = 334 (20.2%)	**HD 22**, N = 243 (14.7%)	**HD 30**, N = 162 (9.79%)
A	Creatinine	0.9 ± 0.4 (0.3 - 2.0)	0.8 ± 0.4 (0.3 - 2.0)	0.9 ± 0.5 (0.3 - 2.0)	1.1 ± 0.5 (0.3 - 2.0)	1.1 ± 0.4 (0.4 - 2.0)
B	Creatinine	1.0 ± 0.4 (0.3 - 2.0)	0.7 ± 0.4 (0.3 - 2.0)	0.7 ± 0.4 (0.3 - 2.0)	0.6 ± 0.3 (0.3 - 2.0)	0.5 ± 0.3 (0.3 - 1.6)
C	Creatinine	0.9 ± 0.4 (0.3 - 2.0)	0.9 ± 0.6 (0.3 - 2.0)	1.0 ± 0.6 (0.3 - 2.0)	0.9 ± 0.6 (0.3 - 2.0)	1.2 ± 0.6 (0.3 - 2.0)
D	Creatinine	0.9 ± 0.4 (0.3 - 2.0)	0.6 ± 0.3 (0.3 - 2.0)	0.6 ± 0.3 (0.3 - 2.0)	0.5 ± 0.2 (0.3 - 2.0)	0.5 ± 0.3 (0.3 - 1.6)

Figure S1. Trajectory of Creatinine for all patients by Hospital days

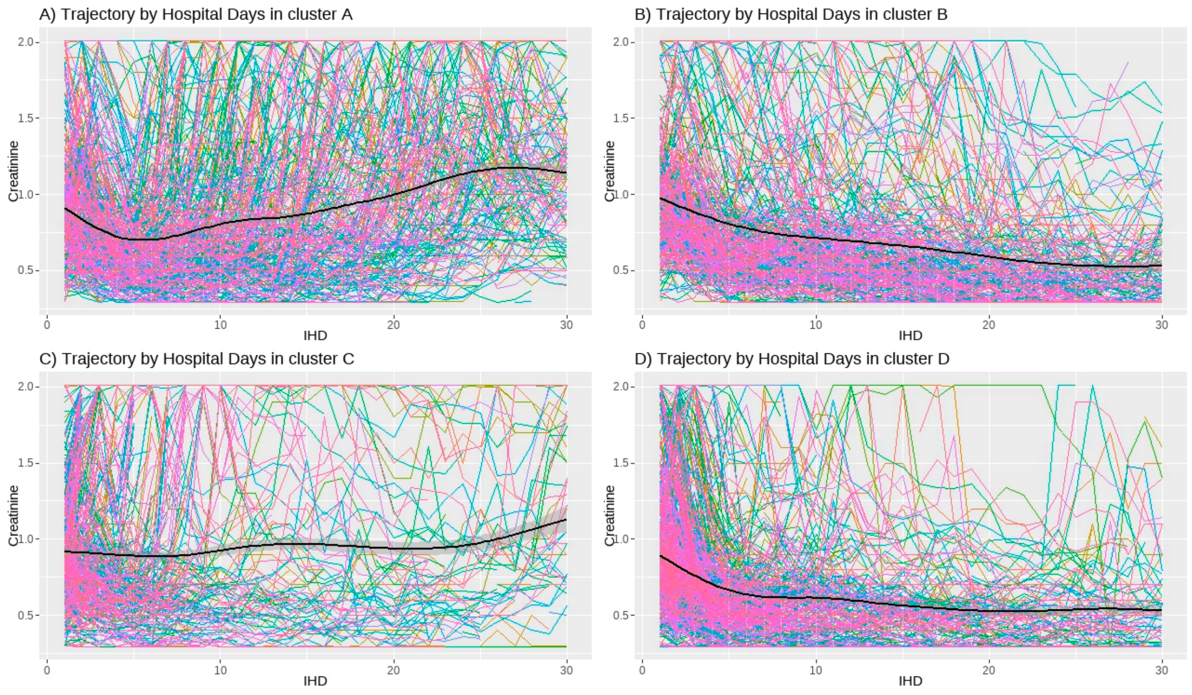

A) Trajectory in cluster A, B) Trajectory in cluster B, C) Trajectory in cluster C, D) Trajectory in cluster D

Table S2. Adjusted Odds Ratios for AKI Based on Various Predictors Controlling for Patient and Disease Factors

Clusters	Predictors	Adjusted odd (95%CI) [a]	p-value	Prevalence
Cluster A				
	Albumin	0.408 (0.275~0.593)	<0.001 **	83.4% (383/459)
	PT	3.361 (1.915~6.411)	<0.001 **	78.3% (246/314)
	RDW	1.998 (1.369~3.007)	<0.001 **	83.5% (385/461)
	Creatinine	2.210 (1.390~3.701)	0.001 *	83.2% (376/452)
	Cystatin C	2.796 (1.534~5.776)	0.002 *	69.5% (155/223)
	eGFR cys	0.507 (0.300~0.819)	0.008 *	70.6% (144/204)
	TB	1.700 (1.177~2.641)	0.010 *	80.9% (317/392)
	WBC	0.734 (0.530~1.005)	0.056	83.5% (385/461)
	eGFR cr	0.730 (0.457~1.126)	0.166	84.9% (220/259)
	pH	0.782 (0.547~1.107)	0.169	83.8% (378/451)
	BUN	1.267 (0.909~1.803)	0.174	83.2% (376/452)
	Platelet	0.866 (0.654~1.152)	0.319	83.5% (385/461)
	Lactate	0.877 (0.607~1.267)	0.482	81.0% (307/379)
Cluster B				
	Albumin	0.314 (0.233~0.417)	<0.001 **	57.7% (259/449)
	PT	1.815 (1.324~2.546)	<0.001 **	47.3% (166/351)
	Creatinine	1.585 (1.194~2.141)	0.002 *	57.7% (259/449)
	WBC	0.754 (0.605~0.936)	0.011 *	58.0% (262/452)
	eGFR cr	0.737 (0.531~1.002)	0.057	62.3% (147/236)
	pH	0.781 (0.589~1.031)	0.083	57.4% (253/441)
	Platelet	0.820 (0.652~1.028)	0.086	58.0% (262/452)
	Lactate	0.886 (0.684~1.144)	0.355	53.9% (214/397)

Clusters	Predictors	Adjusted odd (95%CI) [a]	p-value	Prevalence
	RDW	1.105 (0.847~1.448)	0.464	58.0% (262/452)
	eGFR cys	0.903 (0.637~1.277)	0.564	39.1% (107/274)
	BUN	0.934 (0.717~1.215)	0.608	57.5% (257/447)
	Cystatin C	1.096 (0.749~1.605)	0.634	39.1% (115/294)
	TB	0.954 (0.764~1.188)	0.671	54.3% (226/416)
Cluster C				
	Albumin	0.399 (0.309~0.512)	<0.001 **	51.6% (370/717)
	Creatinine	2.586 (1.951~3.526)	<0.001 **	51.5% (369/717)
	eGFR cr	0.418 (0.280~0.599)	<0.001 **	53.2% (197/370)
	Platelet	0.673 (0.553~0.816)	<0.001 **	51.7% (377/729)
	PT	1.637 (1.284~2.109)	<0.001 **	44.9% (262/583)
	pH	0.640 (0.493~0.829)	<0.001 **	52.6% (367/698)
	BUN	1.532 (1.189~1.987)	0.001 *	51.5% (369/716)
	Cystatin C	1.670 (1.166~2.431)	0.006 *	38.3% (181/472)
	TB	1.365 (1.076~1.748)	0.012 *	49.5% (337/681)
	Lactate	1.415 (1.079~1.859)	0.012 *	49.5% (321/648)
	RDW	1.288 (1.041~1.594)	0.020 *	51.7% (377/729)
	eGFR cys	0.698 (0.504~0.959)	0.028 *	39.1% (174/445)
	WBC	0.956 (0.766~1.188)	0.688	51.7% (377/729)
Cluster D				
	Albumin	0.365 (0.297~0.446)	<0.001 **	41.5% (397/956)
	PT	1.749 (1.383~2.229)	<0.001 **	34.6% (282/815)
	Platelet	0.732 (0.617~0.866)	<0.001 **	41.8% (402/961)
	Lactate	0.748 (0.622~0.894)	0.002 *	38.0% (329/866)
	RDW	1.320 (1.102~1.582)	0.003 *	41.9% (403/962)
	Creatinine	1.327 (1.087~1.625)	0.006 *	41.5% (397/956)

Clusters	Predictors	Adjusted odd (95%CI)[a]	p-value	Prevalence
	Cystatin C	1.443 (1.100~1.891)	0.008 *	26.5% (184/694)
	eGFR cys	0.752 (0.589~0.958)	0.022 *	27.0% (174/645)
	WBC	0.826 (0.700~0.973)	0.023 *	41.8% (402/961)
	TB	1.206 (1.004~1.449)	0.044 *	39.0% (357/915)
	eGFR cr	0.856 (0.703~1.037)	0.115	41.9% (248/592)
	pH	0.896 (0.733~1.095)	0.284	41.1% (379/922)
	BUN	1.080 (0.892~1.307)	0.425	41.5% (396/955)

[a] All adjusted odds ratios were calculated controlling for age, TBSA, inhalation injury, ARDS, sepsis, and the use of ventilators and CRRT.

** Indicates P-value < 0.001. * Indicates P-value < 0.05

Table S3. NRI for Predictors of AKI by Cluster

Clusters	Predictors	NRI (95%CI)	p-value	Prevalence
Cluster A				
	Cystatin C	0.854 (0.669~1.039)	<0.001 **	155/223 (69.5%)
	PT	0.764 (0.560~0.968)	<0.001 **	246/314 (78.3%)
	eGFR cys	0.533 (0.334~0.732)	<0.001 **	144/204 (70.6%)
	Albumin	0.401 (0.163~0.640)	<0.001 **	383/459 (83.4%)
	TB	0.374 (0.143~0.605)	0.002 *	317/392 (80.9%)
	RDW	0.079 (-0.167~0.325)	0.528	385/461 (83.5%)
	Creatinine	Reference		376/452 (83.2%)
Cluster B				
	PT	0.261 (0.114~0.408)	<0.001 **	262/583 (44.9%)
	Albumin	0.169 (0.024~0.315)	0.022 *	370/717 (51.6%)
	Creatinine	Reference		369/717 (51.5%)

Clusters	Predictors	NRI (95%CI)	p-value	Prevalence
	WBC	-0.490 (-0.630~-0.350)	<0.001 **	377/729 (51.7%)
Cluster C				
	Albumin	0.717 (0.542~0.892)	<0.001 **	259/449 (57.7%)
	PT	0.614 (0.441~0.787)	<0.001 **	166/351 (47.3%)
	Cystatin C	0.256 (0.126~0.386)	<0.001 **	115/294 (39.1%)
	Lactate	0.202 (0.008~0.397)	0.042 *	214/397 (53.9%)
	eGFR cys	0.194 (0.076~0.311)	0.001 *	107/274 (39.1%)
	eGFR cr	0.048 (-0.199~0.295)	0.702	147/236 (62.3%)
	Creatinine	Reference		259/449 (57.7%)
	BUN	-0.300 (-0.485~-0.116)	0.001 *	257/447 (57.5%)
	RDW	-0.280 (-0.463~-0.096)	0.003 *	262/452 (58.0%)
	pH	-0.192 (-0.380~-0.003)	0.046 *	253/441 (57.4%)
	Platelet	-0.191 (-0.376~-0.005)	0.044 *	262/452 (58.0%)
	TB	-0.100 (-0.289~0.089)	0.298	226/416 (54.3%)
Cluster D				
	Albumin	0.764 (0.645~0.882)	<0.001 **	397/956 (41.5%)
	PT	0.439 (0.319~0.560)	<0.001 **	282/815 (34.6%)
	Cystatin C	0.188 (0.101~0.274)	<0.001 **	184/694 (26.5%)
	eGFR cys	0.167 (0.079~0.254)	<0.001 **	174/645 (27.0%)
	Lactate	0.156 (0.028~0.285)	0.017 *	329/866 (38.0%)
	TB	0.051 (-0.072~0.174)	0.417	357/915 (39.0%)
	Creatinine	Reference		397/956 (41.5%)
	WBC	-0.070 (-0.198~0.059)	0.289	402/961 (41.8%)
	Platelet	-0.008 (-0.137~0.120)	0.899	402/961 (41.8%)
	RDW	-0.003 (-0.132~0.125)	0.962	403/962 (41.9%)

** Indicates P-value < 0.001. * Indicates P-value < 0.05

Table S4. IDI for Predictors of AKI by Cluster

Clusters	Predictors	IDI (95%CI)	p-value	Prevalence
Cluster A				
	Cystatin C	0.173 (0.140~0.206)	<0.001 **	155/223 (69.5%)
	eGFR cys	0.141 (0.105~0.176)	<0.001 **	144/204 (70.6%)
	PT	0.136 (0.099~0.172)	<0.001 **	246/314 (78.3%)
	TB	0.035 (0.009~0.061)	0.009 *	317/392 (80.9%)
	Albumin	0.035 (-0.004~0.073)	0.076	383/459 (83.4%)
	RDW	0.003 (-0.028~0.034)	0.855	385/461 (83.5%)
	Creatinine	Reference		376/452 (83.2%)
Cluster B				
	Albumin	0.004 (-0.018~0.026)	0.716	370/717 (51.6%)
	Creatinine	Reference		369/717 (51.5%)
	WBC	-0.060 (-0.077~-0.043)	<0.001 **	377/729 (51.7%)
	PT	-0.005 (-0.025~0.016)	0.651	262/583 (44.9%)
Cluster C				
	Albumin	0.125 (0.091~0.159)	<0.001 **	259/449 (57.7%)
	PT	0.049 (0.025~0.073)	<0.001 **	166/351 (47.3%)
	Cystatin C	0.028 (0.001~0.055)	0.044 *	115/294 (39.1%)
	eGFR cys	0.021 (-0.008~0.049)	0.154	107/274 (39.1%)
	Creatinine	Reference		259/449 (57.7%)
	TB	-0.023 (-0.037~-0.008)	0.002 *	226/416 (54.3%)
	BUN	-0.020 (-0.034~-0.006)	0.004 *	257/447 (57.5%)
	RDW	-0.018 (-0.031~-0.005)	0.005 *	262/452 (58.0%)
	eGFR cr	-0.013 (-0.033~0.007)	0.195	147/236 (62.3%)
	Platelet	-0.013 (-0.027~0.001)	0.062	262/452 (58.0%)

Clusters	Predictors	IDI (95%CI)	p-value	Prevalence
	Lactate	-0.009 (-0.025~0.008)	0.298	214/397 (53.9%)
	pH	-0.009 (-0.022~0.005)	0.228	253/441 (57.4%)
Cluster D				
	Albumin	0.095 (0.075~0.115)	<0.001 **	397/956 (41.5%)
	PT	0.029 (0.015~0.042)	<0.001 **	282/815 (34.6%)
	Cystatin C	0.019 (0.005~0.033)	0.008 *	184/694 (26.5%)
	eGFR cys	0.015 (-0.001~0.030)	0.061	174/645 (27.0%)
	Lactate	0.011 (-0.000~0.021)	0.052	329/866 (38.0%)
	Platelet	0.006 (-0.003~0.014)	0.191	402/961 (41.8%)
	RDW	0.002 (-0.006~0.010)	0.705	403/962 (41.9%)
	Creatinine	Reference		397/956 (41.5%)
	WBC	-0.003 (-0.011~0.005)	0.404	402/961 (41.8%)
	TB	-0.002 (-0.009~0.006)	0.650	357/915 (39.0%)

** Indicates P-value < 0.001. * Indicates P-value < 0.05

Table S5. Net Benefit Comparison for AKI Prediction by Decision Curve Analysis

Clusters	Strategy	Threshold_0.3	Threshold_0.35	Threshold_0.4	Threshold_0.45	Threshold_0.5	Threshold_0.55	Threshold_0.6	Threshold_0.65	Threshold_0.7	Threshold_0.75	Threshold_0.8	Threshold_0.85	Threshold_0.9	Threshold_0.95
Cluster A															
	Cystatin C	0.576 (0.505~0.648)	0.576 (0.502~0.648)	0.540 (0.463~0.616)	0.529 (0.449~0.607)	0.514 (0.433~0.595)	0.483 (0.397~0.568)	0.461 (0.372~0.547)	0.433 (0.340~0.522)	0.397 (0.299~0.491)	0.374 (0.270~0.473)	0.374 (0.270~0.470)	0.335 (0.218~0.443)	0.380 (0.322~0.440)	0.346 (0.289~0.407)
	PT	0.592 (0.517~0.665)	0.571 (0.494~0.648)	0.534 (0.453~0.616)	0.503 (0.416~0.588)	0.497 (0.412~0.582)	0.492 (0.402~0.578)	0.466 (0.370~0.560)	0.432 (0.327~0.534)	0.387 (0.272~0.497)	0.380 (0.262~0.494)	0.346 (0.213~0.471)	0.371 (0.236~0.488)	0.279 (0.109~0.424)	0.285 (0.071~0.433)
	eGFR cys	0.599 (0.527~0.669)	0.561 (0.485~0.636)	0.547 (0.470~0.624)	0.532 (0.449~0.612)	0.508 (0.422~0.593)	0.518 (0.433~0.601)	0.458 (0.368~0.546)	0.434 (0.340~0.525)	0.399 (0.295~0.498)	0.341 (0.227~0.448)	0.385 (0.282~0.481)	0.361 (0.258~0.455)	0.374 (0.316~0.435)	0.313 (0.258~0.370)
	Albumin	0.568 (0.490~0.644)	0.559 (0.477~0.638)	0.538 (0.452~0.622)	0.520 (0.430~0.607)	0.469 (0.374~0.562)	0.429 (0.330~0.527)	0.397 (0.287~0.503)	0.385 (0.271~0.493)	0.335 (0.207~0.456)	0.313 (0.175~0.441)	0.257 (0.094~0.403)	0.212 (0.022~0.379)	0.184 (-0.052~0.385)	0.279 (0.073~0.428)
	TB	0.566 (0.488~0.642)	0.544 (0.460~0.625)	0.514 (0.427~0.601)	0.505 (0.415~0.594)	0.492 (0.398~0.584)	0.471 (0.371~0.567)	0.436 (0.328~0.539)	0.396 (0.284~0.505)	0.376 (0.253~0.494)	0.302 (0.161~0.437)	0.313 (0.168~0.449)	0.324 (0.165~0.467)	0.223 (0.023~0.395)	0.022 (-0.324~0.307)
	Creatinine	0.551 (0.471~0.630)	0.532 (0.447~0.614)	0.512 (0.423~0.599)	0.511 (0.420~0.601)	0.480 (0.383~0.578)	0.443 (0.339~0.545)	0.416 (0.306~0.526)	0.381 (0.265~0.496)	0.354 (0.225~0.479)	0.279 (0.132~0.420)	0.196 (0.029~0.357)	0.279 (0.123~0.420)	0.335 (0.187~0.461)	0.341 (0.284~0.399)
	Treat All	0.537 (0.454~0.617)	0.502 (0.412~0.588)	0.460 (0.363~0.554)	0.411 (0.305~0.513)	0.352 (0.236~0.464)	0.280 (0.151~0.405)	0.190 (0.045~0.331)	0.074 (-0.092~0.235)	-0.080 (-0.273~0.107)	-0.296 (-0.528~-0.071)	-0.620 (-0.910~-0.339)	-1.160 (-1.547~-0.785)	-2.240 (-2.820~-1.678)	-5.480 (-6.641~-4.355)
Cluster B															
	Cystatin C	0.171 (0.114~0.229)	0.142 (0.089~0.196)	0.138 (0.087~0.189)	0.120 (0.070~0.170)	0.108 (0.061~0.155)	0.094 (0.047~0.141)	0.097 (0.051~0.142)	0.099 (0.057~0.141)	0.098 (0.058~0.137)	0.108 (0.077~0.140)	0.104 (0.074~0.136)	0.104 (0.074~0.136)	0.104 (0.074~0.136)	0.104 (0.074~0.136)
	eGFR cys	0.178 (0.122~0.235)	0.146 (0.092~0.200)	0.136 (0.086~0.186)	0.101 (0.053~0.149)	0.108 (0.062~0.155)	0.100 (0.054~0.145)	0.097 (0.054~0.139)	0.091 (0.049~0.132)	0.099 (0.063~0.135)	0.104 (0.074~0.136)	0.104 (0.074~0.136)	0.104 (0.074~0.136)	0.104 (0.074~0.136)	0.104 (0.074~0.136)

Clusters	Strategy	Threshold_0.3	Threshold_0.35	Threshold_0.4	Threshold_0.45	Threshold_0.5	Threshold_0.55	Threshold_0.6	Threshold_0.65	Threshold_0.7	Threshold_0.75	Threshold_0.8	Threshold_0.85	Threshold_0.9	Threshold_0.95
	PT	0.165 (0.107~0.225)	0.142 (0.081~0.205)	0.113 (0.052~0.174)	0.081 (0.024~0.138)	0.077 (0.019~0.137)	0.054 (-0.005~0.114)	0.071 (0.014~0.129)	0.068 (0.012~0.124)	0.050 (-0.009~0.107)	0.069 (0.012~0.124)	0.073 (0.015~0.128)	0.068 (0.002~0.125)	0.108 (0.077~0.140)	0.104 (0.074~0.136)
	Albumin	0.163 (0.104~0.223)	0.132 (0.074~0.192)	0.106 (0.045~0.167)	0.080 (0.017~0.145)	0.081 (0.015~0.148)	0.065 (-0.003~0.135)	0.056 (-0.013~0.127)	0.030 (-0.047~0.109)	0.023 (-0.062~0.108)	0.054 (-0.028~0.133)	0.093 (0.019~0.160)	0.081 (0.003~0.149)	0.058 (-0.038~0.137)	0.112 (0.080~0.144)
	Creatinine	0.135 (0.069~0.201)	0.103 (0.035~0.172)	0.051 (-0.020~0.124)	0.009 (-0.066~0.083)	-0.008 (-0.087~0.070)	0.016 (-0.058~0.091)	0.004 (-0.074~0.080)	0.032 (-0.039~0.105)	0.071 (0.008~0.136)	0.081 (0.019~0.140)	0.089 (0.028~0.143)	0.102 (0.050~0.149)	0.116 (0.083~0.148)	0.104 (0.074~0.136)
	Treat All	0.084 (0.014~0.157)	0.014 (-0.062~0.092)	-0.068 (-0.150~0.016)	-0.165 (-0.255~-0.073)	-0.282 (-0.381~-0.181)	-0.424 (-0.534~-0.312)	-0.602 (-0.726~-0.476)	-0.831 (-0.972~-0.687)	-1.136 (-1.301~-0.968)	-1.564 (-1.761~-1.362)	-2.205 (-2.451~-1.952)	-3.273 (-3.602~-2.936)	-5.409 (-5.903~-4.904)	-11.819 (-12.806~-10.808)
Cluster C															
	Albumin	0.231 (0.189~0.275)	0.218 (0.174~0.263)	0.207 (0.162~0.254)	0.200 (0.155~0.248)	0.189 (0.141~0.238)	0.168 (0.119~0.219)	0.150 (0.096~0.203)	0.135 (0.078~0.192)	0.122 (0.062~0.181)	0.093 (0.027~0.157)	0.088 (0.018~0.155)	0.142 (0.090~0.192)	0.140 (0.112~0.168)	0.103 (0.079~0.128)
	PT	0.233 (0.190~0.276)	0.224 (0.180~0.267)	0.212 (0.167~0.257)	0.201 (0.155~0.248)	0.199 (0.152~0.245)	0.184 (0.136~0.232)	0.165 (0.115~0.214)	0.158 (0.108~0.207)	0.145 (0.095~0.195)	0.145 (0.094~0.195)	0.100 (0.043~0.155)	0.084 (0.028~0.137)	0.120 (0.094~0.147)	0.100 (0.076~0.125)
	Cystatin C	0.242 (0.201~0.284)	0.235 (0.194~0.278)	0.226 (0.185~0.268)	0.217 (0.176~0.260)	0.201 (0.158~0.243)	0.178 (0.134~0.222)	0.173 (0.128~0.218)	0.169 (0.123~0.215)	0.181 (0.137~0.224)	0.164 (0.121~0.207)	0.145 (0.102~0.187)	0.131 (0.092~0.167)	0.120 (0.094~0.147)	0.103 (0.078~0.128)
	Lactate	0.229 (0.186~0.274)	0.218 (0.174~0.263)	0.212 (0.167~0.258)	0.200 (0.153~0.247)	0.181 (0.131~0.230)	0.172 (0.120~0.224)	0.162 (0.108~0.215)	0.143 (0.085~0.200)	0.103 (0.040~0.165)	0.113 (0.049~0.174)	0.135 (0.079~0.189)	0.164 (0.135~0.195)	0.113 (0.087~0.139)	0.100 (0.076~0.125)
	eGFR cys	0.238 (0.197~0.281)	0.229 (0.188~0.271)	0.220 (0.178~0.262)	0.211 (0.169~0.253)	0.199 (0.156~0.240)	0.181 (0.137~0.225)	0.172 (0.125~0.217)	0.162 (0.116~0.210)	0.169 (0.124~0.214)	0.181 (0.143~0.221)	0.150 (0.107~0.191)	0.147 (0.118~0.176)	0.125 (0.099~0.152)	0.103 (0.078~0.128)
	Creatinine	0.228 (0.184~0.274)	0.222 (0.178~0.268)	0.223 (0.178~0.269)	0.213 (0.165~0.260)	0.199 (0.150~0.247)	0.182 (0.133~0.232)	0.164 (0.112~0.215)	0.142 (0.086~0.196)	0.127 (0.068~0.185)	0.125 (0.063~0.184)	0.154 (0.097~0.210)	0.104 (0.029~0.171)	0.088 (0.015~0.154)	0.123 (0.096~0.150)
	Treat All	0.090 (0.034~0.147)	0.020 (-0.041~0.081)	-0.062 (-0.127~0.004)	-0.159 (-0.230~-0.086)	-0.275 (-0.353~-0.195)	-0.416 (-0.503~-0.327)	-0.593 (-0.691~-0.493)	-0.821 (-0.932~-0.707)	-1.124 (-1.254~-0.991)	-1.549 (-1.705~-1.389)	-2.186 (-2.382~-1.987)	-3.248 (-3.509~-2.982)	-5.373 (-5.763~-4.974)	-11.745 (-12.527~-10.947)
Cluster D															

Clusters	Strategy	Threshold_0.3	Threshold_0.35	Threshold_0.4	Threshold_0.45	Threshold_0.5	Threshold_0.55	Threshold_0.6	Threshold_0.65	Threshold_0.7	Threshold_0.75	Threshold_0.8	Threshold_0.85	Threshold_0.9	Threshold_0.95
	Albumin	0.076 (0.048~0.106)	0.054 (0.026~0.083)	0.042 (0.015~0.071)	0.036 (0.008~0.065)	0.031 (0.003~0.061)	0.015 (-0.015~0.044)	0.014 (-0.017~0.045)	0.019 (-0.013~0.050)	0.012 (-0.022~0.046)	0.011 (-0.025~0.047)	0.021 (-0.016~0.055)	0.036 (0.003~0.065)	0.059 (0.044~0.075)	0.059 (0.044~0.075)
	PT	0.062 (0.034~0.091)	0.069 (0.042~0.098)	0.052 (0.026~0.078)	0.043 (0.019~0.068)	0.038 (0.014~0.062)	0.044 (0.021~0.068)	0.045 (0.023~0.068)	0.054 (0.033~0.075)	0.054 (0.035~0.073)	0.056 (0.036~0.076)	0.057 (0.038~0.077)	0.061 (0.046~0.077)	0.059 (0.044~0.075)	0.059 (0.044~0.075)
	Cystatin C	0.070 (0.047~0.095)	0.066 (0.044~0.088)	0.063 (0.044~0.084)	0.064 (0.045~0.084)	0.059 (0.042~0.077)	0.055 (0.039~0.072)	0.058 (0.043~0.075)	0.056 (0.040~0.072)	0.059 (0.044~0.075)	0.059 (0.044~0.075)	0.059 (0.044~0.075)	0.059 (0.044~0.075)	0.059 (0.044~0.075)	0.059 (0.044~0.075)
	eGFR cys	0.067 (0.045~0.092)	0.072 (0.050~0.094)	0.066 (0.046~0.087)	0.065 (0.047~0.084)	0.062 (0.046~0.080)	0.055 (0.039~0.072)	0.059 (0.044~0.075)	0.059 (0.044~0.075)	0.059 (0.044~0.075)	0.059 (0.044~0.075)	0.059 (0.044~0.075)	0.059 (0.044~0.075)	0.059 (0.044~0.075)	0.059 (0.044~0.075)
	Lactate	0.058 (0.027~0.089)	0.042 (0.013~0.072)	0.033 (0.003~0.064)	0.035 (0.007~0.063)	0.044 (0.018~0.072)	0.036 (0.011~0.060)	0.051 (0.031~0.072)	0.050 (0.032~0.069)	0.051 (0.032~0.071)	0.054 (0.037~0.072)	0.059 (0.044~0.075)	0.059 (0.044~0.075)	0.059 (0.044~0.075)	0.059 (0.044~0.075)
	Creatinine	0.021 (-0.011~0.054)	0.019 (-0.011~0.051)	0.031 (0.000~0.062)	0.022 (-0.008~0.054)	0.021 (-0.010~0.053)	0.030 (0.001~0.058)	0.039 (0.012~0.067)	0.044 (0.019~0.068)	0.048 (0.027~0.070)	0.052 (0.033~0.072)	0.059 (0.044~0.075)	0.059 (0.044~0.075)	0.059 (0.044~0.075)	0.059 (0.044~0.075)
	Treat All	-0.063 (-0.105~-0.020)	-0.145 (-0.190~-0.099)	-0.240 (-0.289~-0.190)	-0.353 (-0.406~-0.298)	-0.489 (-0.546~-0.428)	-0.654 (-0.718~-0.587)	-0.861 (-0.933~-0.785)	-1.126 (-1.209~-1.040)	-1.481 (-1.577~-1.380)	-1.977 (-2.093~-1.856)	-2.721 (-2.866~-2.570)	-3.962 (-4.155~-3.760)	-6.443 (-6.732~-6.140)	-13.885 (-14.465~-13.281)
